# Behind the Mask of Parkinsonism: A Case Report and Literature Review on Progressive Supranuclear Palsy

**DOI:** 10.7759/cureus.47313

**Published:** 2023-10-19

**Authors:** Saad Asbeutah, Galina Ponomareva, Meron Molla, Shruti Shah

**Affiliations:** 1 Neurology, Kuwait University, Kuwait City, KWT; 2 Neurology, University College Dublin School of Medicine, Dublin, IRL; 3 Internal Medicine, Pomeranian Medical University, Szczecin, POL; 4 Internal Medicine, Byramjee Jeejeebhoy (BJ) Medical College, Pune, IND

**Keywords:** movement abnormalities, vertical gaze palsy, cognitive impairment, focal-dystonia, postural instability, bradykinesia, neurodegenrative, progressive supranuclear palsy (psp), atypical parkinsonism, parkinsons disease

## Abstract

Progressive supranuclear palsy (PSP) is a neurodegenerative condition that typically emerges in adulthood and does not exhibit any familial inheritance pattern. PSP is characterized by gradual stiffness in the central body, an inability to move the gaze upward voluntarily, postural instability, and a decline in cognitive function linked to frontal lobe dysfunction. Clinical assessment reveals a variety of findings, and cases of PSP frequently go unnoticed or are incorrectly diagnosed as other conditions. Notably, prominent neurotransmitter-related changes in PSP involve damage to the dopaminergic nigrostriatal pathway and cholinergic impairment in multiple regions. We hereby present a case of a 71-year-old female patient whose medical journey unfolds as a perplexing riddle. Despite the collective expertise of several physicians, she found herself bearing the weight of a misdiagnosis ascribed to Parkinson's Disease (PD) erroneously. She initially presented with recurring falls due to postural instability and bradykinesia, which progressed such that she became dependent on a walking aid. A comprehensive physical examination revealed indicators consistent with PSP.

## Introduction

Progressive supranuclear palsy (PSP) is a neurodegenerative disorder that typically emerges in adulthood without any familial pattern. Its most common form, Richardson's syndrome (PSP-RS), is marked by early falls due to postural instability. Additionally, distinct oculomotor signs predominantly affect vertical eye movements, involving slowed saccades that progress into supranuclear gaze palsy [1].

PSP-RS is categorized as a rare ailment, with an incidence rate of approximately five to seven cases per 100,000 individuals [2]. A recent study in the United Kingdom disclosed a zenith prevalence occurring in the age range of 70 to 74, hovering around 18 cases per 100,000 [2]. In contrast, a Japanese study, encompassing an array of PSP phenotypes in conjunction with PSP-RS brought to light an aggregate prevalence of 18 cases per 100,000 across all age strata [3]. Additionally, Yoshida et al. conducted a study on forensic cases, uncovering a higher-than-expected prevalence of early-stage PSP. If these findings apply to broader population-based cohorts, it suggests that PSP may be more common than previously believed [4].

Investigations into external risk factors for PSP have been scarce, and extensive, controlled experiments still need to be done. Lower levels of educational attainment are the only external risk factor consistently supported by multiple studies. Some theories addressing this association are a potential link to diminished “synaptic reserve,” heightened exposure in work or living environments associated with industrial occupations, and lower income levels. Interestingly, factors like residing in rural areas, using well water, exposure to pesticides, and abstaining from smoking, all of which have shown connections with Parkinson's disease (PD) in various studies, do not appear to have any discernible relationship with PSP [5].

PSP is notable for the vast array of anatomical and neurochemical variations across different central nervous system (CNS) areas. Damage to the dopaminergic nigrostriatal pathway and cholinergic impairment in multiple regions consistently appear as the most prominent neurotransmitter-related changes observed [6].

Our case report and extensive literature review on PSP serve the purpose of augmenting the existing scholarly discourse. Through an exhaustive analysis of clinical presentations and diagnostic intricacies, we aspire to provide a nuanced perspective on PSP. By doing so, our intention is to furnish the medical community with additional tools for early recognition and enhanced clinical management of this complex neurological condition.

## Case presentation

A 71-year-old right-handed woman presented at the neurology outpatient office in 2023 with tremors, bradykinesia, vision problems, and a history of multiple falls. She had previously been diagnosed with PD in 2018 when she exhibited a shuffling gait and slow movements.

Four years before her presentation, the patient began experiencing postural instability and multiple falls. Over the last two months, she had more than five falls, totaling 26 falls since the onset of her symptoms. She also mentioned her inability to see the entire staircase, particularly the upper steps, prompting her family to install an elevator in the house to reduce the frequency of her falls. Her postural instability necessitated the use of a walking aid for ambulation.

Three years before her presentation, the patient developed bradykinesia, which progressively worsened over the last few months. Her movements significantly slowed, requiring more time to complete daily tasks. She also experienced multiple freezing episodes while attempting to stand up from a seated position. Additionally, she lost her sense of smell and suffered from slow chewing and difficulty swallowing solids and liquids. She had been prescribed carbidopa/levodopa 25/100 mg for several months, but it proved ineffective, and her condition continued to progress.

Two years prior to her presentation, the patient complained of tremors, characterized by rhythmic, oscillatory movements during purposeful and goal-directed activities, such as reaching for a glass of water. Initially, they manifested as slight shaking in her hands when performing tasks requiring precision, such as writing. Over time, they became more pronounced and noticeable, significantly affecting her ability to perform daily activities independently. The combination of postural instability, visual disturbances, and tremors resulted in her inability to drive.

One year before her presentation, the patient’s family complained of gradual cognitive decline. The family noticed signs of forgetfulness, such as misplacing items and struggling to remember appointments. Her caretaker pointed out her recent behavior of spending large sums of money on international trips with her “new friends” as unlike the patient’s usual behavior.

**Figure 1:**
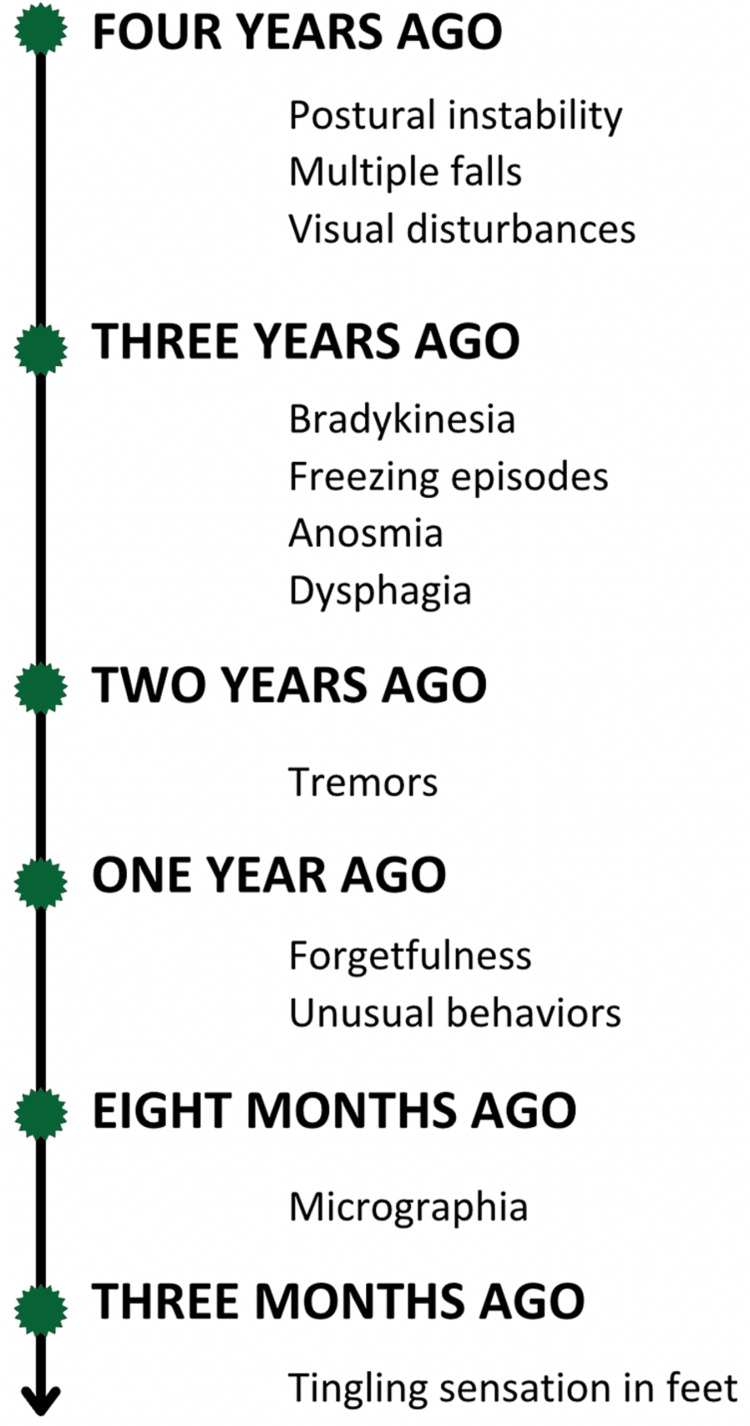
Chronological order of symptoms

Eight months before her presentation, the patient experienced micrographia, leading to illegible handwriting. Three months before her presentation, the patient complained of a tingling sensation in both her feet. This tingling sensation, described as either pins-and-needles or numbness, was progressive in nature. According to the patient's detailed history, the sensation was more pronounced during prolonged standing or walking periods, which led to discomfort and a significant decline in her quality of life. This tingling sensation was accompanied by a sense of weakness in the affected feet, adding a layer of complexity to her motor function. Figure 1 illustrates the chronological progression of the patient's symptoms over time, providing a visual representation of how her medical condition evolved.

Relevant comorbidities included hypothyroidism, diabetes mellitus, moderate obstructive sleep apnea, irritable bowel syndrome, hypercholesterolemia, and gastroesophageal reflux disease. Her family history was remarkable for meningioma in her aunt and brain cancer and dementia in her sister, but no other family members were diagnosed with neurocognitive disorders. She was a nonsmoker and consumed alcohol occasionally.

On physical examination, the patient had normal sitting, standing, and supine blood pressure values of 135/73, 122/85, and 126/76 mmHg, respectively. The patient was morbidly obese (BMI 40). The patient was alert and oriented to person and place but not time. Her Montreal Cognitive Assessment (MoCA) score declined from 25/30 to 22/30 in the span of two months, which indicated mild cognitive impairment. She demonstrated a delay in response time, and conversations were marked by reduced, slow speech output, and a lack of inflection. The patient exhibited short steps with minimal hand swing. Tandem gait and rapid alternating movements were challenging. Additionally, positive Myerson's and glabellar tap signs were present. Episodes of sudden uncontrollable and inappropriate laughter, which indicated a pseudobulbar state, were also noted. Resting tremors were not observed, and the Romberg, retropulsion, and pronator drift tests all yielded negative results. On examination of cranial nerves, limited vertical gaze was observed, particularly on downward gaze, which indicated a vertical gaze palsy. The presence of vertical gaze palsy was particularly evident in tracking objects. The horizontal gaze was preserved, but voluntary blinking in general presented difficulty. Her face was stiff and immobile with a reduction in facial expression, which indicated dystonia. Increased rigidity of the trunk was noted, especially on standing. Muscle strength in the lower limbs was graded 4 out of 5 according to the Medical Research Council (MRC) scale and reflexes were graded +2. The remainder of the neurological exam was unremarkable.

The patient's presentation, characterized by vertical gaze palsy, postural instability, bradykinesia, dysphagia, hypophonia, and cognitive impairment, combined with the patient's medical history and a lack of response to carbidopa/levodopa, strongly suggested a diagnosis of PSP. A series of investigations were performed to exclude other potential causes. 

**Figure 2:**
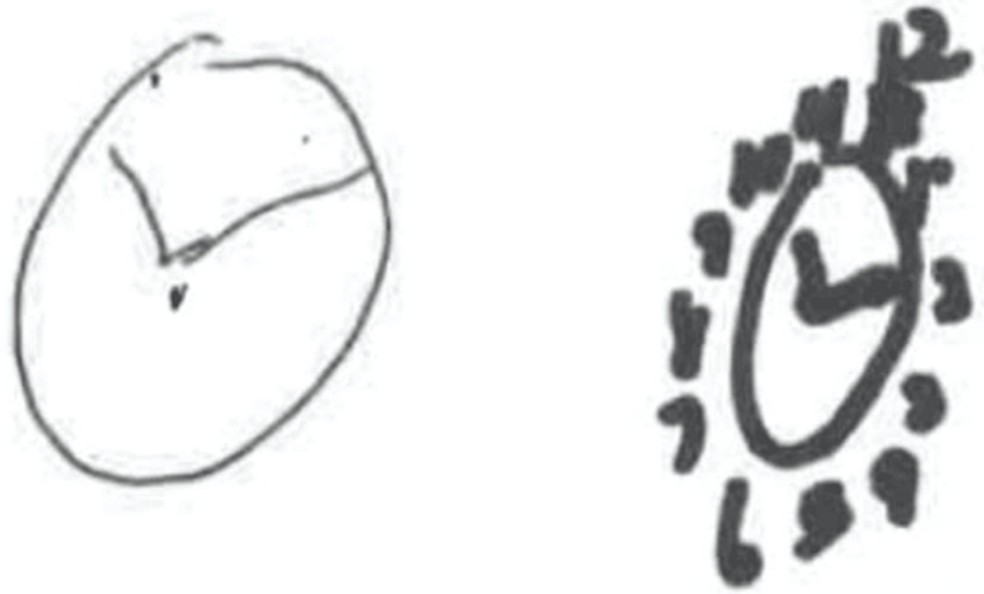
Drawing of a clock. Left: June 2023, Right: August 2023

Her comprehensive metabolic panel and complete blood count were unremarkable. Carotid Doppler ultrasound results demonstrated anterograde and normal flow in the vertebral artery, according to the North American Symptomatic Carotid Endarterectomy Trial (NASCER). Multiplanar and multisequence magnetic resonance imaging (MRI) of the brain was performed before and after intravenous contrast. Images were obtained from a 1.5 Tesla closed MRI and 7.5 mL of Gadavist intravenous contrast was administered. No acute infarct, intracranial hemorrhage, or mass lesion was noticed. The ventricles, sulci, and cisterns were all normal in size, shape, and position for age. There were no intra- or extra-axial masses or fluid collections and no abnormalities of the orbits, thalami, midbrain, or brainstem. The MRI of the cervical spine revealed chronic degenerative changes in C3-C4 and C4-C5 discs. Autonomic nervous system function was tested with a TM flow to evaluate the tingling sensation in her lower limbs. The TM flow demonstrated small fiber neuropathy with autonomic dysfunction. We also assessed her handwriting and drawings of a clock (Figure 2) over a three-month period, which demonstrated notable micrographia and lack of precision.

After conducting a thorough examination of the patient's clinical presentation and reviewing the investigation findings, it became clear that her collection of symptoms met the criteria for probable PSP.

## Discussion

PSP is the most common degenerative form of atypical parkinsonism with characteristic features of early postural instability with falls; akinesia/parkinsonism; oculomotor deficits, marked by slowing of vertical saccades followed by a vertical gaze palsy; frontal lobe impairments, characterized by speech and language problems; and behavioral changes in a patient who is 40 years old or older. While living, the diagnosis of PSP is based on clinical features. No laboratory or imaging studies are diagnostic. Imaging can be supportive if there is predominant midbrain atrophy on MRI, but the absence of this feature does not rule out the diagnosis. A levodopa trial in patients with parkinsonism and suspected PSP can assist with the diagnosis; poor or unsustained response to levodopa therapy is generally observed in patients with PSP and can help to distinguish PSP from idiopathic PD. Neuropathologic examination remains the gold standard for its definitive diagnosis. The pathologic diagnosis of PSP is based on identifying a high density of neurofibrillary tangles and neuropil threads in the basal ganglia and brainstem [7].

The 2017 Movement Disorder Society (MDS-PSP) diagnostic criteria include basic features, core features, supportive features, and operational definitions. The basic features (mandatory inclusion criteria, mandatory exclusion criteria,context-dependent exclusion criteria) apply to all probable, possible, and suggestive criteria. Core clinical features are defined by their functional domain (ocular motor dysfunction (O), postural instability (P), akinesia (A), and cognitive dysfunction (C)) and stratified by presumed levels of certainty (1 (highest), 2 (mid), 3 (lowest)) they contribute to the diagnosis of PSP. Supportive clinical clues (CC) include levodopa-resistance, hypokinetic, spastic dysarthria, dysphagia, and photophobia.

Derived from a combination of core clinical features and CCs, it proposes four levels of diagnostic certainty (Table 1) [8]. According to the MDS-PSP criteria, our patient initially qualified for probable PSP with predominant parkinsonism (PSP-P). Still, her symptoms eventually met probable PSP with predominant frontal presentation (PSP-F) and probable PSP-RS criteria.

**Table 1:** 2017 MDS-PSP Criteria

Diagnostic Certainty	Definition	Combinations	Predominance Type
Definite PSP	Gold standard defining the disease entity	Neuropathological diagnosis	Any clinical presentation
Probable PSP	Highly specific, but not very sensitive for PSP	(O1 or O2) + (P1 or P2)	PSP with Richardson’s syndrome
		(O1 or O2) + A1	PSP with progressive gait freezing
		(O1 or O2) + (A2 or A3)	PSP with predominant parkinsonism
		(O1 or O2) + C2	PSP with predominant frontal presentation
Possible PSP	Substantially more sensitive, but less specific for PSP	O1	PSP with predominant oculomotor dysfunction
		O2 + P3	PSP with Richardson’s syndrome
		A1	PSP with progressive gait freezing
		(O1 or O2) + C1	PSP with predominant speech/language disorder
		(O1 or O2) + C3	PSP with predominant corticobasal syndrome
Suggestive PSP	Suggestive of PSP, but not passing the threshold for possible or probable PSP	O2 or O3	PSP with predominant oculomotor dysfunction
		P1 or P2	PSP with predominant postural instability
		O3 + (P2 or P3)	PSP with Richardson’s syndrome
		(A2 or A3) + (O3, P1, P2, C1, C2, CC1, CC2, CC3, or CC4)	PSP with predominant parkinsonism
		C1	PSP with predominant speech/language disorder
		C2 + (O3 or P3)	PSP with predominant frontal presentation
		C3	PSP with predominant corticobasal syndrome

A PD misdiagnosis initially challenged the diagnosis of PSP in our patient and further challenged the level of subclassification, as her symptoms met the criteria for several PSP subtypes. Given these diagnostic difficulties, we reviewed the literature to gain more insight into the variety of PSP presentations. 

Owolabi et al. reported a case of PSP in an 82-year-old man with symptoms of postural instability, vertical gaze palsy, and resistance towards anti-parkinsonian treatments. The patient was initially misdiagnosed with PD and eventually found to have PSP, supported by the characteristic “hummingbird sign” on brain MRI. Our patient similarly presented with episodes of freezing, downward gaze palsy, postural instability, and rigidity and was misdiagnosed with PD. PSP was only considered after a carbidopa/levodopa treatment trial proved ineffective. Although she had thus far not demonstrated any classic changes on MRI, their case and ours reflect a reality in which PSP often gets misdiagnosed as PD, especially in the early stages [9]. 

Similarly challenging is the presentation of PSP alongside comorbidities that present with neurological findings. Ikram et al. presented a case of a 54-year-old man with symptoms of bilateral facial weakness, involuntary movements, sensory disturbances, and dysphagia. Physical examination corroborated these neurological abnormalities, including slowed eye movements and reduced sensation. Our patient similarly presented with dysphagia, downward gaze palsy, and peripheral neuropathy. However, her sensory disturbances were attributed to small fiber neuropathy associated with diabetes. The two cases demonstrate the challenge of teasing out the true origin of specific neurological symptoms [10].

Atypical motor symptoms may also present alongside the typical PSP pattern. Janati et al. reported the first case of torticollis in a 76-year-old male patient with PSP, indicating a possible link to pathologic changes in the striatum and brainstem. Their patient also had blepharospasm and dysfluency of the extrapyramidal type before developing torticollis. Their report made a strong case for these three focal dystonias sharing a common pathophysiological mechanism. While our patient does not present with torticollis or blepharospasm, the stiffness of her facial muscles and progressive dysphagia does indicate focal dystonias of the muscles of facial expression and muscles of swallowing [11]. 

While PSP criteria primarily focus on neurological signs that affect movement, neuropsychiatric effects may also be present alongside motor and neurocognitive changes, proving yet another layer of complexity for diagnosis. Madhusoodanan et al. discussed the diagnostic challenges of psychiatric symptoms in early-stage PSP. Their study reported the case of a patient initially diagnosed with Alzheimer’s dementia and mood disorder, who later displayed hypersexual behavior and cognitive symptoms associated with PSP. Subsequent examination revealed an unsteady gait and an absent vertical gaze. While our patient showed only mild cognitive impairment, her behavioral changes, presenting as unprovoked laughter and unusually indulgent spending, suggest the onset of neuropsychiatric changes [12].

Another consideration prompted by neuropsychiatric symptoms is the use of dopaminergic medications. Kita et al. reported a case of probable PSP in a 64-year-old male patient with chronic schizophrenia who was on treatment with antipsychotics for about 13 years. Drug-induced parkinsonism was suspected when he started exhibiting bradykinesia, upper limb rigidity, dysphagia, short-term memory impairment, photophobia, falls, and other extrapyramidal symptoms, and the antipsychotics were tapered off. Despite that, he showed persistent parkinsonism symptoms. Eventually, he met the requirements for PSP-RS based on the MDS-PSP criteria with moderate cognitive impairment (MMSE: 16/30). His diagnostic imaging was also consistent with PSP [13]. 

On the other hand, Garcia reported a case of an older male patient whose PSP pattern fit the PSP-RS criteria but was complicated by a prior psychiatric background. After the PSP diagnosis, the patient developed a premorbid personality, cognitive rigidity, dichotomous thinking, impulsiveness, depression, lack of tolerance to physical limitations, passive-aggressive episodes, and notable hygienic and dietary changes. Notably, the patient was partially responsive to antidepressants but improved further with the discontinuation of carbidopa/levodopa and reduced carbidopa/levodopa/entacapone dose. Garcia concluded that psychotic symptoms may be present alongside motor PSP symptoms. Furthermore, it was suggested that levodopa/carbidopa may be detrimental to the neuropsychiatric condition, an essential consideration for those with psychiatric comorbidities [14].

While neuropsychiatric comorbidities increase the complexity of PSP diagnosis, neurocognitive assessments may be helpful in elucidating patterns that point towards PSP and away from neuropsychiatric disease. Spaccavento et al. highlighted a case of atypical PSP in a 72-year-old male patient with a dementia syndrome dominated by progressive apraxia of speech and aphasia. Similar to our patient, their patient also demonstrated supranuclear vertical gaze palsy, reduced facial mimicry, balance deficits leading to frequent falls, and moderate cognitive impairment (MMSE 20/30). However, their patient demonstrated mood disturbances, such as anxiety, irritability, and depression, and widespread cortical atrophy on MRI [15].

Berthier et al. presented a case of two patients, aged 71 and 73, who initially developed progressive speech and language decline before developing motor deficiencies. The results of a battery of tests assessing language production, reception, and memory demonstrated that both patients exhibited dynamic aphasia, a speech and language deficit of language production, and increased disinhibition, characterized by echolalia. Our study echoed their findings, wherein the patient demonstrated some level of disinhibition with increased financial spending and spontaneous laughter. The authors argued that understanding the nature of the language deficits in PSP would not only improve the diagnostic criteria for the condition. Still, they may also serve to anatomically delineate the areas of deficit leading to cognitive impairment associated with PSP [16].

Another investigation on episodic memory impairment in PSP was conducted by Macedo et al., which compared neurocognitive changes between healthy controls and patients with neuropsychiatric disorders. Their systematic review of 38 studies showed that PSP patients generally have lower scores in both verbal and visual episodic memory tests compared to healthy controls. While there were no noteworthy differences in serial memory performance between PSP and PD or other atypical parkinsonism disorders, patients with PSP performed better than those with Alzheimer’s disease in memory tests, indicating a more severe memory impairment in Alzheimer’s. This important adverse finding helps further delineate the neurocognitive patterns associated with PSP [17].

However, some studies demonstrate that the patterns thus far attributed to PSP may have relatively easy and fast boundaries. Although PSP typically occurs after the age of 40, Megherbi et al. reported an atypical probable PSP case in a 35-year-old male patient with gait disturbances, postural instability leading to falls, axial rigidity, vertical gaze palsy, dysphagia, and a slight frontal syndrome. They ruled out other differential diagnoses, including Wilson’s disease and anti-DPPX encephalitis. Since onset after age 40 is one of the diagnostic requirements, they encouraged consideration for early presentations similar to their patient [18].

Kurz et al. presented a case of autopsy-confirmed PSP that did not meet the criteria for probable PSP until very late in the patient’s disease process, even after alternative diagnoses had been ruled out. The patient initially presented with postural instability, hypokinesia, hypomimia, dysarthria, and backward falls but did not develop supranuclear gaze palsy until 11 years from onset. In addition, the patient also presented with an exceptionally long disease duration of 15 years. They encouraged reassessing the diagnostic criteria for PSP, given that at least 18% of cases do not present with supranuclear gaze palsy until late, and between 9% and 59% of patients do not develop supranuclear gaze palsy at all [19]. Table 2 condenses the salient points from all previously discussed case report summaries, placing a spotlight on the primary symptoms in each case.

**Table 2:** Summary of primary signs and symptoms in aforementioned PSP case reports Information obtained from [9-16] and [18,19]

Case	Primary Signs and Symptoms
Owolabi et al. (2013)	Postural instability, vertical gaze palsy and resistance towards anti-parkinsonian treatments
Ikram et al. (2021)	Bilateral facial weakness, involuntary movements, sensory disturbances and dysphagia
Janati et al. (1986)	Torticollis, blepharospasm and dysfluency of the extrapyramidal type
Madhusoodanan et al. (2014)	Hypersexual behavior, unsteady gait and an absent vertical gaze
Kita et al. (2022)	Bradykinesia, upper limb rigidity, dysphagia, short-term memory impairment, photophobia and falls
Garcia (2017)	Cognitive rigidity, dichotomous thinking, impulsiveness, depression, lack of tolerance to physical limitations, passive-aggressive episodes and hygienic and dietary changes
Spaccavento et al. (2014)	Progressive apraxia of speech and aphasia, supranuclear vertical gaze palsy, reduced facial mimicry, balance deficits leading to frequent falls and moderate cognitive impairment
Berthier et al. (2021)	Progressive speech and language decline and dynamic aphasia
Megherbi et al. (2021)	Postural instability, gait disturbances, axial rigidity, vertical gaze palsy, dysphagia and a slight frontal syndrome.
Kurz et al. (2016)	Postural instability, hypokinesia, hypomimia, dysarthria, and backward falls and supranuclear gaze palsy 11 years after

Currently, a PSP diagnosis can only be confirmed based on neuropathological findings at autopsy [7]. However, given the importance of the neuropathological conclusions, an argument could be made for using imaging as a robust diagnostic factor rather than a contributory finding. Despite differing presentations, a common thread in the studies mentioned above demonstrates the importance of neuroimaging for PSP diagnosis. The case of Owolabi et al. established how brain imaging can aid in diagnosing PSP by using the classic “hummingbird sign” and characteristic features of midbrain atrophy on brain MRI [9]. Spaccavento et al. used brain MRI to corroborate their clinical findings by demonstrating widespread cortical atrophy [15]. Berthier et al. argued that understanding the nature of the language deficits in PSP alongside MRI imaging may also anatomically outline areas of deficit that result in some of the neurocognitive changes seen with PSP [16]. Macedo et al. used neuroimaging findings that demonstrated the involvement of strict frontal structures in PSP episodic memory dysfunction [17]. Finally, Kita et al. showed that PSP may, again, be differentiated from other Parkinsonian syndromes through the revelation of mesencephalic tegmental atrophy and reduced bilateral striatal uptake on brain MRI and 123i-ioflupane SPECT scans [13].

Likewise, Choi et al. found a correlation between oculomotor dysfunction and superior cerebellar peduncle atrophy, which was not present in patients with PD, providing a diagnostic feature that could be used to differentiate PSP from PD. However, as negative imaging does not rule out the diagnosis, as with our patients, clinical signs remain at the center of successful diagnosis. To diagnostically clarify the differences between PSP subtypes, Choi et al. primarily investigated oculomotor changes, clinical features, and brain atrophy measures associated with different PSP subtypes in 123 patients. They found that patients with PSP-RS had more severe oculomotor degenerative changes early in their disease progress when compared with patients with PSP-P and PSP-PGF. They suggested that the degree of oculomotor degeneration could be used as a diagnostic tool to classify a patient’s PSP subtype in the early stages of the disease. In addition, they found an association between the degree of oculomotor dysfunction and the presence of clinical features - indicating that grading the severity of clinical symptoms may also facilitate PSP subclassification. Because our patient fit the criteria for all three subtypes mentioned above but did not demonstrate any changes on MRI, she could benefit from this type of assessment were it to be employed for diagnostic purposes [20].

Incorporating our collective clinical experience, the application of robust evidence, and a comprehensive review of the existing literature, we provided expert recommendations on diagnostic approaches for PSP. Early and accurate diagnosis of PSP had posed a formidable challenge, given its diverse clinical presentations and the overlap of symptoms with other neurodegenerative disorders. However, our review underscored the significance of employing a multidisciplinary diagnostic strategy. This included integrating detailed clinical assessments, neuroimaging techniques like MRI, and genetic testing where appropriate. Timely referrals to specialized movement disorder clinics had also proven invaluable in confirming a PSP diagnosis. We emphasized the importance of a thorough clinical evaluation that encompassed a comprehensive medical history, detailed neurological examination, and standardized rating scales. As part of the evaluation process, awareness of the recently updated diagnostic criteria, such as the MDS criteria for PSP, had significantly enhanced diagnostic accuracy. Our expert recommendations aimed to guide healthcare professionals in navigating the challenging landscape of PSP diagnosis, ultimately improving the chances of early intervention and enhanced patient care.

While this case report provides valuable insights into the diagnosis and management of probable PSP, it is essential to acknowledge its limitations. Firstly, our findings are based on a single patient, which may limit the generalizability of the conclusions to a broader population. Additionally, the type of pen used to evaluate our patient's handwriting was different from her usual writing instrument, which might have influenced the legibility of her handwriting. Nevertheless, despite these limitations, this case underscores the complexity of diagnosing and managing PSP, shedding light on the need for further research in this area.

## Conclusions

PSP, characterized as a neurodegenerative condition, has frequently been associated with misdiagnoses, thus underscoring the imperative for more refined diagnostic methodologies. During the era under examination, diagnostic criteria predominantly leaned on clinical patterns, substantiated by neuroimaging findings. The initial presentation of our patient notably exhibited symptoms that bore a striking resemblance to PD, culminating in an extended diagnostic odyssey. Throughout our comprehensive literature review, we unveiled the intricate tapestry of comorbidities and the extensive spectrum of clinical presentations, both of which posed formidable challenges to the clinical diagnosis of PSP. While neuroimaging served as a pivotal tool in confirmation, contemporary research initiatives have introduced alternative modalities to differentiate PSP from other neurodegenerative syndromes. Our expert recommendations, informed by the amalgamation of clinical expertise, robust empirical evidence, and a meticulous review of the literature, ardently endorse the adoption of a multidisciplinary diagnostic paradigm for PSP. By facilitating prompt referrals to specialized clinics, conducting clinical evaluations, and maintaining an awareness of the latest diagnostic criteria, we aspire to expedite early interventions and fortify the standard of patient care.
